# Does complementary and alternative medicine (CAM) use reduce negative life impact of headaches for chronic migraineurs? A national survey

**DOI:** 10.1186/s40064-016-2362-7

**Published:** 2016-07-07

**Authors:** Jieun Lee, Amrita Bhowmick, Amy Wachholtz

**Affiliations:** Psychology Department, Chung-Ang University, 84 Heukseok-ro, Dongjak-gu, Seoul, Korea; VP Community Development, Health Union LLC, 1 International Plaza, Philadelphia, PA 19113 USA; Department of Psychology, University of Colorado Denver, Denver, CO 80204 USA

**Keywords:** Chronic migraineurs, CAM treatment satisfaction, Psychiatric comorbidities, Migraine outcomes, Negative life impact

## Abstract

**Background:**

Chronic migraine is a disabling condition that impacts multiple aspects of migraineurs’ lives. Although pharmacological treatments can help to treat the pain associated with migraine headache, chronic migraineurs often experience side-effects of pharmacological treatments. Those experiences may contribute to the observed growth in complementary and alternative medicine (CAM) use among migraineurs. Relatively little is known about the patterns of CAM treatment and the characteristics of chronic migraineurs. Therefore, the purpose of the present study is to investigate the characteristics of chronic migraineurs who use CAM treatment and the relationship among satisfaction with current CAM use, negative life impact, migraine outcomes, and psychiatric comorbidities among chronic migraineurs.

**Methods:**

2907 participants were recruited from a well-known online migraine headache resource. All participants were US adults aged 18 years or older. Migraineurs are referred to this website through various routes (e.g., referral from healthcare providers, internet search, obtaining information from research papers, personal invitation from other users, and information shared on social media etc.). Participants completed a 30-min self-report-survey in the spring of 2014.

**Results:**

Almost half of the participants reported that they are currently using more than three different CAM treatments even though the majority of the participants reported neutral or dissatisfied with their current CAM treatment. Chronic migraineurs who use CAM treatments were more likely to experience prolonged or frequent migraine headaches (p = .018, η^2^ = .0021), and experience greater negative life impact from their headaches (p = .000, η^2^ = .0172) compared to non-CAM users. CAM treatment satisfaction was inversely related to the number of psychiatric comorbidities, frequency of migraines, and number of migraine symptoms (p’s < .05). However, CAM treatment satisfaction was more strongly correlated with migraine outcomes than psychiatric comorbidities.

**Conclusions:**

Chronic migraineurs often pursue multiple CAM treatments in spite of low levels of satisfaction with those treatments. Patients who experience relief from traditional treatments are less likely to seek the out additional CAM treatments. Thus it is often the sicker migraine patients who use CAM. More attention is needed to consider migraine treatment resistance, and psychological factors in planning the treatment of chronic migraineurs as those factors may play an important role in treatment choices by patients.

## Background

Migraine headaches are a common health condition which affects more than 10 % of the global adult population (Adams et al. 2013). In the US, 14.2 % of US adults were affected by migraine or severe headaches (Burch et al. 2015). Migraine is a disabling condition that impacts not only productivity and attendance at work or school, but also quality of life at home.

Pharmacological treatments of migraine headaches can help to relieve the pain and symptoms associated with migraine headache. However, migraineurs often experience side-effects of pharmacological treatments and frequent use of medications can lead to medication overuse headaches (Adams et al. 2013). The limits to pharmacological treatments may explain high usage of complementary and alternative medicine (CAM) among migraineurs (Wells et al. 2011). The number of migraineurs who are using CAM treatment in conjunction with traditional medical treatments has been growing over the years (Eisenberg et al. 1998; Jacobson et al. 2009; Kaptchuck and Eisenberg 1998). Consistent with this pattern, a number of studies investigating the prevalence and patterns of CAM treatment in migraineurs have been slowly growing, however, previous studies were conducted within individual headache clinics with limited populations (Adams et al. 2013; Wells et al. 2011).

Relatively little is known about the patterns of CAM treatment and the characteristics of migraineurs in the general community; therefore, the current study focuses on migraineurs who are recruited nationwide from across the US. Due to technological advancement and widespread internet access in recent years, most people search for health-related information on the internet and also seek emotional and instrumental support from the internet community. Considering this trend, the present study analyzed the data collected from a community-based website for migraine headache in the US.

Previous study (Wachholtz et al. 2015) found that chronic migraineurs often experienced high levels of dissatisfaction with medical and CAM treatments for migraine headaches. This study further indicated that chronic migraineurs tended to seek multiple treatments instead of focusing on a single treatment. This tendency may occur because migraineurs use CAM treatments not only to treat their migraine symptoms but also to improve the quality of life by preventing headaches or by increasing their energy levels (Wells et al. 2011). Previous studies (Lipton et al. 2003; Malone et al. 2015; Smitherman et al. 2011; Wachholtz et al. 2015) indicated that migraine headache was associated with negative impacts in different domains of life (e.g., physical function, social function, role function, mental health). However, relatively little is known about the relationship between satisfaction with CAM use and the negative life impacts of migraine among migraineurs.

In order to address gaps in previous studies mentioned above, the present study investigated the data collected on the web-community in order to provide insights into (1) the characteristics of chronic migraine sufferers, (2) chronic migraineurs who use CAM treatment compared to who do not use CAM treatment; (3) the prevalence of different types of CAM use; and (4) the details of negative life impact affected by migraine; and (5) the relationship among satisfaction with current CAM use, negative life impact, migraine outcomes, and psychiatric comorbidities among chronic migraineurs.

## Methods

### Participants

2907 participants were recruited from a well-known online migraine headache resource (Migraine in America, website: https://migraine.com/). All participants were US adults aged 18 years or older have been diagnosed with chronic migraine by their physicians who are diagnosed chronic migraine using ICD-9 criteria. However, 197 participants did not complete the survey or did not meet criteria of being diagnosed with chronic migraine; these individuals were excluded in the analysis of the present study. Among 2710 completing participants, the majority of participants were female (92.8 %; shown in Table 1). More than half of the participants were older than 40 years old. Most of the participants (75 %) experienced their first migraine symptoms more than 10 years ago and 25 % of the respondents reported experiencing migraine symptoms lasting more than 4 h at least 20 times per month. Regarding symptoms associated with migraine, head pain and sensitivity to light were most common, followed by sensitivity to sound, difficulty concentrating, nausea, and mood change (Table 1). The most common comorbid disorders associated with migraine were depression and anxiety, followed by chronic pain, irritable bowel syndrome, and chronic fatigue. Almost 70 % of the participants experienced at least one psychiatric comorbiditity. The most commonly reported triggers to migraine headaches were stress and environmental triggers, followed by lack of sleep (Table 2). Almost 70 % of the respondents reported taking special steps to avoid triggers while only 34 % of the participants reported keeping a journal to track their migraine episodes. Lastly, more than half of the participants either avoided or stopped migraine medications due to side effects (Table 2).

**Table 1:** Patient demographics, and migraine characteristics

	N	%
Gender		
Female	2514	92.8
Male	196	7.2
Age in years		
<18	0	0
18–24	189	7
25–39	914	33.7
40–54	1294	47.7
>55	313	11.5
First migraine symptoms		
<1 year	38	1.4
1–5 years	267	9.9
6–10 years	348	12.8
10+ years	2057	75.9
Migraine symptoms frequency lasting 4+ hours per month		
1–4 times	464	17.1
5–9 times	564	20.8
10–14 times	513	18.9
15–19 times	488	18
20+ times	681	25.1
Symptoms associated with migraine headache		
Head pain	2552	94.2
Sensitivity to light	2374	87.6
Nausea and/or vomiting	2047	75.5
Diarrhea, constipation	786	29
Difficulty concentrating	2090	77.1
Fatigue	1941	71.6
Neck pain	1877	69.3
Dizziness/lightheadedness	1588	58.6
Sensitivity to sound	2172	80.1
Visual changes	1391	51.3
Weakness	1190	43.9
Mood change	1601	59.1
Sensitivity to smell	1650	60.9
Numbness/tingling	908	33.5
Vertigo	761	28.1
Puffy eyelid	608	22.4
Food cravings	542	20
Other	381	14.1

**Table 2:** Triggers to migraine and side effects of migraine medications

	N	%
Triggers to migraine		
Stress	1557	57.5
Environmental (weather etc.)	1589	58.6
Lack of sleep	1382	51
Hormones/menstrual cycle	1179	43.5
Certain food or drinks	1254	46.3
Missing meals	1096	40.4
Certain smells	1058	39
Alcohol/drugs	709	26.2
Physical activity	603	22.3
Sexual activity	165	6.1
Other	392	14.5
Any special steps to avoid these triggers	1793	66.2
Currently keep a journal to track migraine episodes	937	34.6
*Avoided* using a medicine due to *side effects*	1689	62.3
*Stopped* using a medicine due to *side effects*	1709	63.1
Side effects		
Nausea/vomiting	613	22.6
Stomach ache	363	13.4
Rebound headaches	898	33.1
Dizziness	594	21.9
Other	1237	45.6

### Procedures

All methods were approved by the University of Massachusetts IRB and all participants indicated their consent to participate in the study prior to answering any of the study questions. Migraineurs are referred to this website through various routes (e.g., referral from healthcare providers, internet search, obtaining information from research papers, personal invitation from other users, and information shared on social media etc.). IP address logging prevented participants from responding to the survey multiple times. Participants were not compensated in any way for their participation in the survey. Participants completed a 30-min self-report-survey in the spring of 2014.

## Materials

The survey included questions regarding demographics, migraine symptoms and diagnosis, general impact of migraine, and medications and treatments of migraine headache. A subset of questions was selected for analysis in the present study: demographics (e.g., gender, age), questions related to migraine onset, frequency of headache symptoms, comorbid disorders, triggers to migraine, and headache treatments (both conventional and CAM). Participants indicated which CAM therapies they were currently using or had historically used from an extensive list. The present study used CAM definition adopted by Cochrane Collaboration, which defined CAM as “all practices and ideas self-defined by their users as preventing or treating illness or promoting health and well-being” (Zollman and Vickers 1999, p. 693). Total scores for current CAM treatments were created by adding the numbers of therapies each participant selected. For those who selected “other” participants were asked to specify the type of therapies they are currently using and each response was assigned into different categories of current CAM use (i.e., bodywork, mental well-being, Eastern-based practices, nutritional therapy, and others). Satisfaction with current pharmacological treatments and CAM treatments was assessed by one question: “how satisfied are you with your current therapies for migraine?” Participants were asked to rate how much they are satisfied on 5-point Likert scale ranging from extremely dissatisfied (coded as 0) to extremely satisfied (coded as 4) for each category of CAM (Table 3). The same scale was applied to satisfaction with pharmacological treatments.

**Table 3:** ANOVA of migraine factors comparing groups on CAM use status

	F(df-btw, df-tot)	p	η^2^
Negative life impact	47.382 (1, 2709)	.000	.0172
Years since first migraine	5.585 (1, 2709)	.018	.0021
Frequency of migraine symptoms lasting 4+	10.290 (1, 2709)	.001	.0038
Number of migraine symptoms	388.026 (1, 2709)	.000	.1253
Number of psychiatric comorbid disorders	74.105(1, 2709)	.000	.0266
Triggers to migraine	1361.729 (1, 2709)	.000	.0555

Negative life impact was measured by 6 statements (1) Migraines affect my ability to maintain relationships; (2) Migraines have impacted my professional achievement; (3) I have lost a job due to my migraines; (4) I’ve lost friends due to my migraines; (5) Migraines contributed to my divorce/separation; and (6) Migraines impact my relationship with my child/children). Total score for negative life impact was a summation of the negative life items endorsed.

### Data analysis

Descriptive statistics, analysis of variance, and Pearson’s R correlation analyses were used to describe participant’s demographic information and migraine characteristics and examine relationships among different variables in the survey data. Path analyses were performed to investigate differential relationships among satisfaction with CAM treatment and pharmacological treatment, number of psychiatric co-morbid disorders, migraine frequency, number of migraine symptoms, and negative life impact. In order to explore the discrepancies in the characteristics of CAM users and non-CAM users, two CAM use groups were created; participants who are using at least one CAM treatment currently was considered as *CAM users* and participants who are not using any CAM treatment currently were classified as *non*-*CAM users*. One-way ANOVA was conducted on different outcomes with two CAM use groups. Three satisfaction groups were created in order to explore how satisfaction with current CAM use impact on negative life impact and frequency of migraine; (1) Satisfied Group, which includes participants answering either highly satisfied or satisfied with their current CAM treatment, (2) Neutral Group, which includes participants reporting neutral satisfaction with current CAM treatment, and (3) CAM Dissatisfaction Group, which includes participants responding either highly dissatisfied or dissatisfied with their current CAM treatment. One-way ANOVAs were performed on different outcomes with three CAM Satisfaction Groups.

## Results

### The characteristics of CAM users versus non-CAM users

**Fig. 1:**
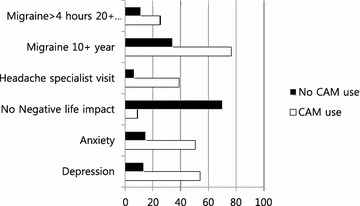
Comparisons of responses (in percent) by CAM use status (N = 2710)

Figure 1 shows that CAM users are more likely to experience prolonged or frequent migraine headaches specifically lasting longer than 4 h for more than 20 days per month, have more years of suffering from migraine headaches, are more likely to visit headache specialist, and experience more negative life impact, and tend to experience depression and anxiety.

One-way ANOVA tests were performed to determine statistical differences in migraine frequency, migraine years, and negative life impact between CAM users and non-CAM users. As shown in Table 4, there was a significant effect of CAM status on negative life impact (p = .000, η^2^ = .0172), years since first migraine (p = .018, η^2^ = .0021), migraine frequency (p = .001, η^2^ = .0038), number of migraine symptoms (p = .000, η^2^ = .1253), number of psychiatric comorbid disorders (p = .000, η^2^ = .0266) and triggers to migraine (p = .000, η^2^ = .0555). These results indicated that CAM users experienced significantly more negative life impact, suffered from migraine lasting more than 4 h for more days per month and for more years, experienced more migraine symptoms and more psychiatric comorbidities, and identified more triggers to migraine compared to non-CAM users.

**Table 4:** Type and numbers of current CAM use and CAM use satisfaction (N = 2477)

Type of CAM therapy	# reporting
*Mental well-being*	
Hypnosis	2
Stress reduction	2
Relaxation/meditation	45
Deep breathing	4
Biofeedback	17
Pet therapy	1
Singing	1
Psychotherapy	1
Total	73
*Bodywork*	
Message	1455
Chiropractic	72
EFT (tapping)	1
Osteopathic	2
Exercise	18
Physical therapy	18
Pressure point	5
TMJ work	1
Cranial sacral therapy	3
Muscle stretches	1
Total	1576
*Eastern-based practices*	
Acupuncture	724
Acupressure	10
Chinese herbs	7
Yoga	17
Cupping	1
Reiki	7
Total	766
*Nutritional therapy*	
Vitamin supplements	2032
Diet	1464
Butterbur	19
Tea	2
Caffeine	11
Total	3528
*Others*	
Aromatherapy	19
Avoid light	2311
Avoid other triggers	14
BenGay/biofreeze	5
Homeopathy	3
Cannabis use	10
Prayer	3
Metaphysical healing	1
Hot/cold therapy	1885
Total	4251

	Number (%)
Current CAM use (number of methods used)	
0	356 (13.1)
1	429 (15.8)
2	632 (23.3)
3	557 (20.6)
4 or more	736 (27.2)
Current CAM use satisfaction	
Strongly dissatisfied and dissatisfied	861 (31.7)
Neutral	991 (36.6)
Strongly satisfied and satisfied	625 (23)

### The prevalence of patterns of CAM use

The prevalence and patterns CAM use were summarized in the Table 4. Each participant was instructed to select CAM treatment that they were currently using and was also allowed to select more than one CAM treatment. According to the Table 4, avoiding light, hot/cold therapy, diet, taking vitamin supplements, acupuncture, chiropractic, and relaxation/meditation were the frequently used CAM treatment among the participants of the present study. Almost half of the participants reported that they are currently using more than three different CAM treatments even though the majority of the participants reported neutral or dissatisfied with their current CAM treatment (Table 4).

### The details of negative life impact affected by migraine headache

As shown in Table 5, nearly half of the participants responded that migraine headache affected various aspect of their life such as their professional advancement, their interpersonal relationships, and their marriage.

**Table 5:** Negative life impact of migraine headaches (N = 2710)

Event	N (%)
Migraine affect my ability to maintain relationships	1027 (37.9 %)
Migraines have impacted professional advancement	1207 (44.5 %)
I have lost a job due to my migraine	612 (22.6 %)
Migraines impact my relationship with my child/children	476 (17.6 %)
I’ve lost friends due to my migraines	183 (6.8 %)
Migraines contributed to my divorce/separation	1029 (38 %)

### The relationship between satisfaction with current CAM use and quality of life and migraine headache

One-way ANOVA tests were performed to see whether there were significant differences in negative life impact and frequency of migraine headache among three CAM satisfaction status groups. Results indicated that there were significant differences in negative life impact (p = .000, η^2^ = .0287), frequency of migraine lasting more than 4 h per month (p = .000, η^2^ = .0550), number of migraine symptoms (p = .000, η^2^ = .0149) and number of psychiatric comorbid disorders (p = .003, η^2^ = .0048) among three groups (shown in Table 6).

**Table 6:** One-way ANOVA in negative life impact, migraine outcomes, and psychiatric comorbid disorders by current CAM Satisfaction Groups

	F(df-btw, df-tot)	p	η^2^
Negative life impact	36.564 (2, 2476)	.000	.0287
Frequency of migraine symptoms lasting 4+	71.965 (2, 2476)	.000	.0550
Number of migraine symptoms	18.643 (2, 2476)	.000	.0149
Number of psychiatric comorbid disorders	5.916 (2, 2476)	.003	.0048

**Fig. 2:**
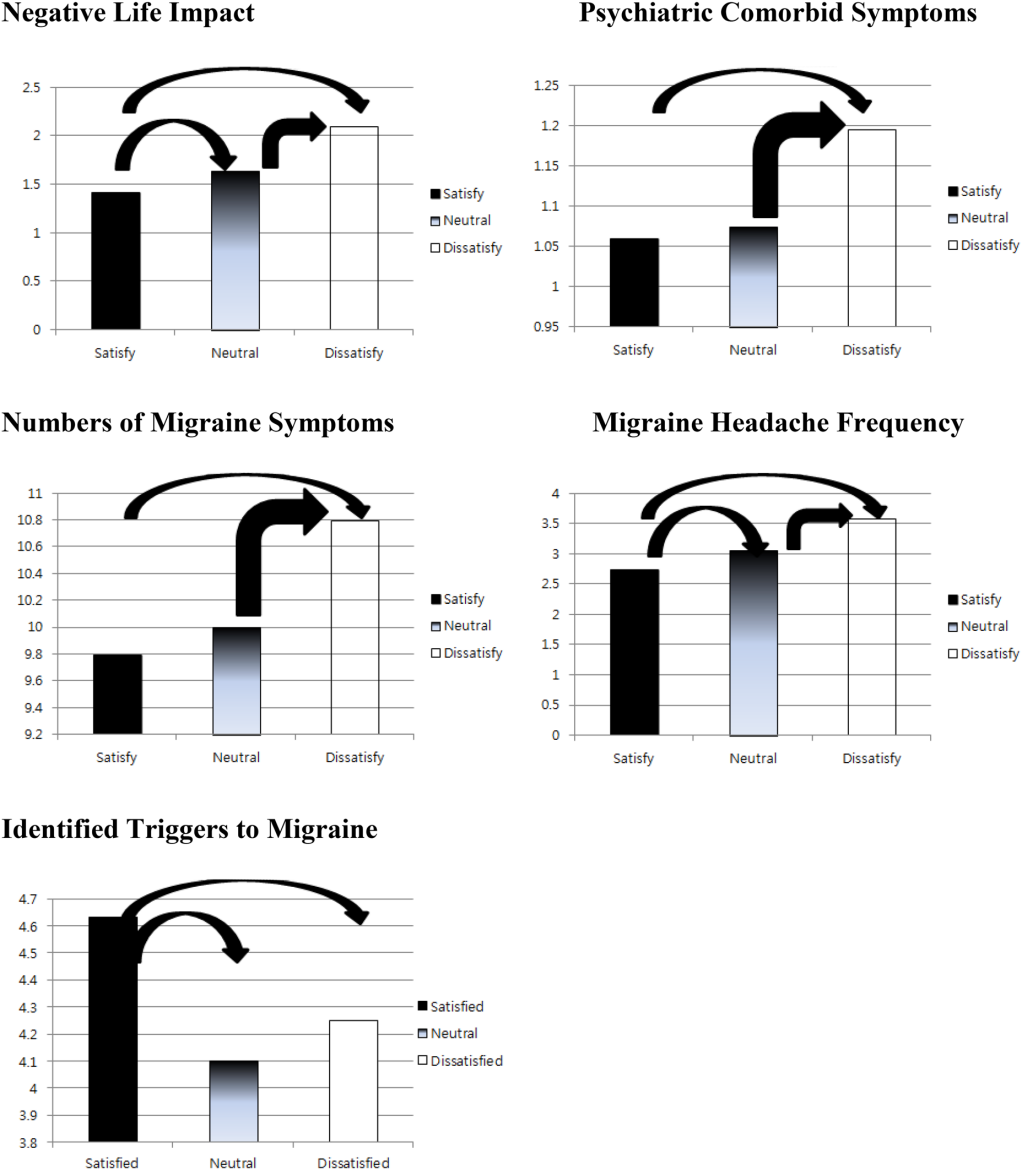
Scheffe test: three satisfaction groups on outcomes. *Note*: *Arrow signs* indicate statistically significant different between two satisfaction groups

In order to further examine group differences, Scheffe post hoc tests were performed. Post-hoc test results revealed that CAM Dissatisfaction Group experienced significantly more negative life impact compared to CAM Neutral Group and CAM Satisfaction Group whereas CAM Neutral Group experienced significantly more negative life impact than CAM Satisfaction Group (Fig. 2). Similar patterns were observed for migraine headache frequency. As shown in Fig. 2, CAM Dissatisfaction Group experienced significantly more frequent migraine headache compared to CAM Neutral Group and CAM Satisfaction Group. There were significant difference between CAM Neutral Group and CAM Satisfaction Group indicating that CAM Neutral Group experience more frequent migraine headaches than CAM Satisfaction Group (Fig. 2).

Number of migraine symptoms and psychiatric comorbid disorders revealed similar patterns; CAM Dissatisfaction Group experienced significantly more migraine symptoms and psychiatric comorbidities compared to CAM Neutral Group and CAM Satisfaction Group whereas there were no significant differences between CAM Neutral Group and CAM Satisfaction Group (Fig. 2). Lastly, as shown in Fig. 2, different pattern was observed on triggers to migraine; CAM Satisfaction Groups identified significantly more triggers than CAM Neutral Group and CAM Dissatisfaction Group whereas no significant difference was observed between CAM Neutral Group and CAM Dissatisfaction Group.

### Path analysis of treatment satisfaction (CAM treatment) on negative life impact of migraine

**Fig. 3:**
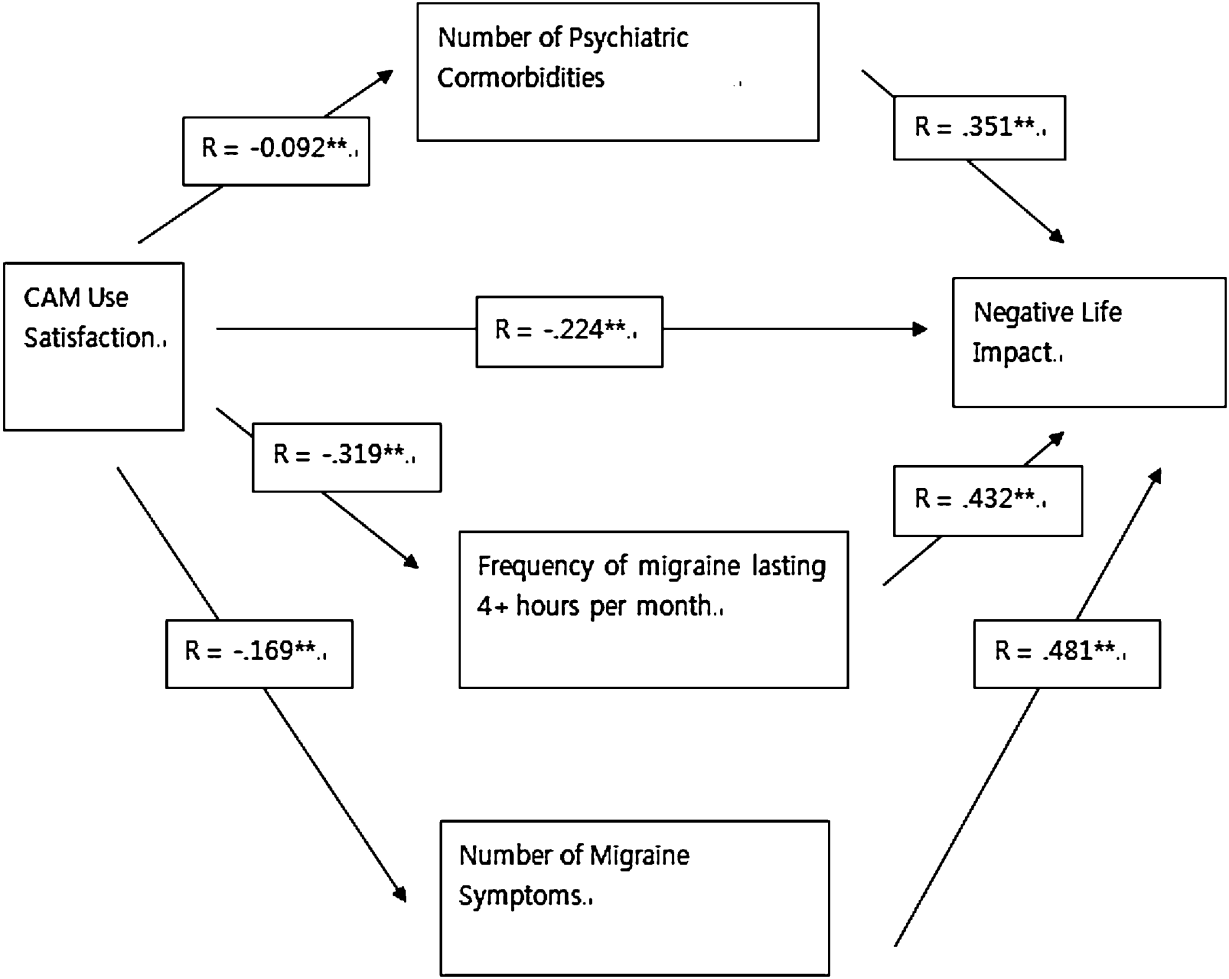
Path analysis of CAM treatment on negative life impact

Figure 3 showed the differential relationships among treatment satisfaction of CAM use, migraine outcomes, psychiatric comorbid disorders, and negative life impact. CAM treatment satisfaction was significantly negatively related to number of psychiatric comorbidities, frequency of migraine, and number of migraine symptoms. CAM treatment satisfaction was more strongly correlated with migraine outcomes (e.g., frequency and migraine symptoms) compared to psychiatric comorbidities. However, migraine outcomes and psychiatric comorbidities were all significantly positively related to negative life impact.

## Discussion

Consistent with previous studies (Adams et al. 2013; Gaul et al. 2009; Lambert et al. 2010; Malone et al. 2015; Rossi et al. 2006; Wachholtz et al. 2015), the present study revealed that CAM users were more likely to have comorbid mental health issues, suffered from more intense headaches for a longer period of time, and experienced more negative life impact of migraine compared to Non-CAM users. These results suggest that individuals may seek out alternative sources of healing when standard biomedical treatments do not meet their needs, are too expensive, considered too dangerous, or the side effects of treatments are too overwhelming. In spite of common use of CAM treatments as the last resort to treat intense intractable pain, migraineurs usually do not inform their medical providers about their CAM use (Lambert et al. 2010; Rossi et al. 2006). This result highlights the importance of investigating the patterns of CAM use among chronic migraineurs. A number of studies (Adams et al. 2013; Gaul et al. 2009; Lambert et al. 2010; Rossi et al. 2006) examined migraineurs’ CAM using patterns but most studies were conducted in headache clinics so the present study investigated the CAM use among chronic migraineurs in the community settings.

According to the current study, managing triggers such as avoiding light and applying hot and cold packs were the most commonly used CAM treatment among chronic migraineurs. Next frequently used CAM treatments include nutritional therapies. These results can be explained by people’s tendency to select the CAM methods that are most easily available when treating migraine symptoms and trying CAM for the first time. More extensive therapies such as massage, acupuncture and chiropractic treatment were also frequently used CAM treatments among the participants of the current study but were less popular than home-based CAM options, which were consistent with the results from previous studies (Gaul et al. 2009; Rossi et al. 2006). These results may suggest important clinical implications for chronic migraineurs. Individuals with chronic migraine may experience difficulty in engaging in more extensive CAM treatments on a consistent basis due to their physical disability associated with migraine and economic costs related to these non-home based CAM practices. However, they may be more willing to try methods they can practice at home such as managing triggers, monitoring their pain and symptoms, and self-pain-management techniques such as meditation/relaxation and activities that enhance mental well-being (e.g., pet therapy, music, prayer). According to the current study, almost half of the chronic migraineurs identified various triggers to their migraine headaches; however, only 35 % of the participants reported keeping a journal to track migraine episodes. Education for tracking migraine episodes will enhance chronic migraineurs’ ability in managing triggers to migraine. Two of the most frequently identified triggers to migraine among chronic migraineurs in the present study were stress and lack of sleep. Those two factors can contribute to the development of other comorbid disorders such as psychological problems and sleep disorders when those become chronic, therefore, it should be important for health care providers to provide sleep hygiene education and make a referral to psychotherapy interventions such as sleep education or stress management therapy when treating chronic migraineurs.

Like most chronic illnesses, migraine headaches profoundly impact individuals’ health as well as his/her family and vocational life. Previous research suggested the impacts of migraine headache on different aspects of life (Lipton et al. 2003; Malone et al. 2015; Smitherman et al. 2011; Wachholtz et al. 2015) and the impact of migraine in physical and social functioning was even greater for migraineurs compared to individuals who struggle with other chronic illnesses (Solomon et al. 1994). The present study revealed that chronic migraine headaches placed similar burden on migraineurs to non-chronic migraineurs as chronic migraine not only causes physical disability but also brings social disability due to its chronicity and breath of symptoms associated with migraine. Thus, a multi-discipline approach in treatment planning can be beneficial in assisting chronic migraineurs. In addition to traditional pharmacological treatments, various CAM treatments such as psychosocial treatments, nutritional treatments, and bodywork therapies can be recommended to chronic migraineurs in order to manage their migraine symptoms.

It should also be noted that migraines may be treatment resistant. The majority of the participants in the present study reported that they suffered from migraine headaches for more than 10 years and either avoided or stopped migraine medications due to side effects. Furthermore, high numbers of chronic migraineurs in the current study reported either neutral or dissatisfaction with either pharmacological treatments or CAM treatments, which was consistent with previous studies (Malone et al. 2015; Wachholtz et al. 2015). In spite of its limited benefit, chronic migraineurs are known to attempt multiple CAM treatments and this treatment-seeking-pattern may occur because (1) traditional pharmacological treatments do not properly address their pain and symptoms associated with migraine; and (2) CAM treatments help chronic migraineurs to reduce negative impact of migraine by improving their physical and mental wellbeing.

Examining the negative effects of migraine in different domains of life is well established in previous research, however, a closer look at the relationship between treatment satisfaction with CAM use and negative life impact by investigating factors affecting both treatment satisfaction with CAM use and negative life impact has not been conducted in previous studies. According to the present study, when migraineurs had greater satisfaction with alternative medicine treatment, they concurrently had lower levels of negative life impact, less frequently suffered from prolonged migraine headaches, experienced less symptoms associated with migraine, and were less likely to experience psychiatric comorbidities. We would postulate that these results occur because “satisfaction” means that migraineurs have experienced some symptom relief with their CAM treatment, where as “dissastified” suggests that they have not experienced relief from CAM treatments, so they are still suffering and may not see an end to their headaches leading to greater psychiatric comorbidities.

Therefore, satisfaction with CAM treatments may play protective factor for negative effects of migraine headache in daily functioning.

In order to further explore the relationship between CAM treatment satisfaction and negative life impact, the present study examined migraine outcomes (e.g., migraine frequency and number of migraine symptoms) and psychiatric comorbidity (e.g., anxiety, depression, bipolar disorder, PTSD etc.) and how those factors are associated with CAM treatment satisfaction and negative life impact. The results of the present study showed that the inverse relationship between CAM treatment satisfaction and psychiatric comorbidities was weaker than the inverse relationship between CAM treatment satisfaction and migraine outcomes although psychiatric comorbidities and migraine outcomes were all strongly associated with greater levels of negative life impact. These results may indicate that CAM treatment may affect mental well-being of chronic migraineurs positively but in a lesser degree compared to the degree that the effectiveness of CAM treatments affects migraine outcomes. One partial explanation for this is that relatively few numbers of CAM treatments identified in the present study were devoted to improving mental health well-being. In the present study, psychiatric and psychological factors may play the important roles as a contributing factor (i.e., stress as one of the most frequently identified triggers to migraine) as well as a precipitating factor (i.e., psychiatric comorbidities were the most common comorbid disorders among chronic migraineurs) to migraine headache. Psychiatric comorbidities may also be a risk factor to negative life experiences. For instance, chronic migraineurs may blame migraine headache for their disabilities when their disabilities are actually due to their psychiatric comorbidities (Wachholtz et al. 2015) or their pain experience caused by migraine may have been amplified due to psychiatric comorbidities. These results are significant because they emphasized the importance of treating psychiatric comorbidities in chronic migraineurs since appropriate care of the psychiatric comorbidities can directly affect quality of life and/or indirectly to increase quality of life by reducing migraine symptoms. Previous study (Pistoia et al. 2013) indicated that combined treatments to target both migraine symptoms and psychological co-morbidities in order to enhance the quality of life for chronic migraineurs are important, however, only few studies (Kleiboer et al. 2014) investigated the effectiveness of psychological treatments on chronic migraineurs.

## Limitations

Despite the contributions of the present study to the field, there are some limitations. Although the present study recruited participants from one major US migraine headache website and have similar demographics to US migraineurs, the participants of the present study may not be representative for all migraineurs as it was self-selected sample, which is the standard method of collecting data in on-line survey study. In addition, the present study measured symptoms associated with migraine as well as the negative life impact caused by migraine headaches. Future studies need to include questionnaires as well as qualitative methods such as individual interviews as well as focus group data in order to provide more detailed pictures of the negative impact of migraine headache as well as the experiences of chronic migraineurs who seek self-care resources on-line.

## Conclusions

The present study contributed to the field by increasing our knowledge about migraine characteristics, CAM use patterns, and the differential relationships among CAM treatment satisfaction, migraine headache outcomes, psychiatric comorbidities, and negative life impact among chronic migraineurs. Chronic migraineurs usually pursue multiple CAM treatments in spite of low levels of satisfaction with their treatments as they tend to suffer from more severe symptoms of migraine and more psychiatric comorbidities without significant improvement from traditional medical treatments. Consistent with previous literature, chronic migraineurs experience a broad range of migraine symptoms and those symptoms negatively affect various aspects of chronic migraineurs’ life. This shows the complex nature of migraine headache compared to other chronic illnesses and therefore calls for multidisciplinary approach to treatment. CAM treatment may play a protective factor against negative life experiences among chronic migraineurs. However, CAM treatments may have a limited benefit for psychiatric comorbidities. More attention is needed to consider psychiatric and psychological factors in planning the treatment of chronic migraineurs as those factors may play an important role in disease process of migraine headache. The implementation of psychosocial interventions for chronic migraineurs, the proper referral system to psychiatric and psychological treatments by treatment providers, and the establishment of clinical guidelines for evidence-based CAM treatments that target mental well-being in the context of headache clinics can enhance the quality of life among chronic migraineurs. In order to develop clinical guidelines of CAM treatments that focus on mental wellbeing as well as psychiatric and psychological treatments, future studies should examine longitudinal changes based on different types of CAM use that target mental well-beings.

## References

[CR1] Adams J, Barbery G, Lui CW (2013). Complementary and alternative medicine use for headache and migraine: a critical review of the literature. J Head Face Pain.

[CR2] Burch R, Lode S, Lode E, Smitherman T (2015). The prevalence and burden of migraine and severe headache in the United States: updated statistics from government health surveillance studies. J Head Face Pain.

[CR3] Eisenberg DM, Davis RB, Ettner SL, Appel S, Wilkey S, Van Rompay M, Kessler RC (1998). Trends in alternative medicine use in the United States, 1990–1997: results of a follow-up national survey. JAMA.

[CR4] Gaul C, Eismann R, Schmidt T, Ma A, Leinisch E, Wiesse T, Ever S, Henkel K, Franz G, Zierz S (2009). Use of complementary and alternative medicine in patients suffering from primary headache disorders. Cephalalgia.

[CR5] Jacobson IG, White MR, Smith TC, Smith B, Wells TS, Gackstetter GD (2009). Self-reported health symptoms and conditions among complementary and alternative medicine users in a large military cohort. Annu Epidemiol.

[CR6] Kaptchuck TJ, Eisenberg DM (1998). The persuasive appeal of alternative medicine. Ann Intern Med.

[CR7] Kleiboer A, Sorbi M, Silfhout M, Kooistra L, Passchier J (2014). Short-term effectiveness of an online behavioral training in migraine self-management: a randomized controlled trial. Behav Res Therapy.

[CR8] Lambert TD, Morrison KE, Edwards J, Clarke CE (2010). The use of complementary and alternative medicine by patients attending a UK headache clinic. Complement Ther Med.

[CR9] Lipton RB, Liberman JN, Kolodner KB, Bigal ME, Dowson A, Stewart WF (2003). Migraine headache disability and health-related quality of life: a population-based case control study from England. Cephalagia.

[CR10] Malone CD, Bhowmick A, Wachholtz AB (2015). Migraine: treatments, comorbidities, and quality of life, in the USA. J Pain Res.

[CR11] Pistoia F, Sacco S, Carolei A (2013). behavioral therapy for chronic migraine. Curr Pain Headache Rep.

[CR12] Rossi P, Lorenzo GD, Malpezzi MG, Faroni J, Cesarino F, Lorenzo CD, Nappi G (2005). Prevalence, pattern and predictors of use of complementary and alternative medicine (CAM) in migraine patients attending a headache clinic in Italy. Cephalagia.

[CR13] Rossi P, Lorenzo GD, Malpezzi MG, Faroni J, Malpezzi MG, Cesarino F, Nappi G (2006). Use of complementary and alternative medicine by patients with chronic tension-type headache: results of a headache clinic survey. Headache.

[CR14] Smitherman TA, McDermott MJ, Buchanan EM (2011). Negative impact of episodic migraine on a university population: quality of life, functional impairment, and comorbid psychiatric symptoms. Headache.

[CR15] Solomon GD, Skobieranda FG, Gragg LA (1994). Does quality of life differ among headache diagnoses? Analysis using the medical outcomes study instrument. Headache.

[CR16] Wachholtz A, Malone C, Bhowmick A (2015). The chronic migraineur and health services: national survey results. Pain Manag Med.

[CR17] Wells RE, Bertisch SM, Buettne C, Phillip RS, McCarthy EP (2011). Complementary and alternative medicine use among adults with migraines/severe headaches. Headache.

[CR18] Zollman C, Vickers A (1999). ABC of complementary medicine: what is complementary medicine?. Br Med J.

